# Differences in Cell Division Rates Drive the Evolution of Terminal Differentiation in Microbes

**DOI:** 10.1371/journal.pcbi.1002468

**Published:** 2012-04-12

**Authors:** João F. Matias Rodrigues, Daniel J. Rankin, Valentina Rossetti, Andreas Wagner, Homayoun C. Bagheri

**Affiliations:** 1 Institute of Evolutionary Biology and Environmental Studies, University of Zurich, Zurich, Switzerland; 2 Institute of Molecular Life Sciences, University of Zurich, Zurich, Switzerland; 3 Swiss Institute of Bioinformatics, University of Zurich, Zurich, Switzerland; Harvard University, United States of America

## Abstract

Multicellular differentiated organisms are composed of cells that begin by developing from a single pluripotent germ cell. In many organisms, a proportion of cells differentiate into specialized somatic cells. Whether these cells lose their pluripotency or are able to reverse their differentiated state has important consequences. Reversibly differentiated cells can potentially regenerate parts of an organism and allow reproduction through fragmentation. In many organisms, however, somatic differentiation is terminal, thereby restricting the developmental paths to reproduction. The reason why terminal differentiation is a common developmental strategy remains unexplored. To understand the conditions that affect the evolution of terminal versus reversible differentiation, we developed a computational model inspired by differentiating cyanobacteria. We simulated the evolution of a population of two cell types –nitrogen fixing or photosynthetic– that exchange resources. The traits that control differentiation rates between cell types are allowed to evolve in the model. Although the topology of cell interactions and differentiation costs play a role in the evolution of terminal and reversible differentiation, the most important factor is the difference in division rates between cell types. Faster dividing cells always evolve to become the germ line. Our results explain why most multicellular differentiated cyanobacteria have terminally differentiated cells, while some have reversibly differentiated cells. We further observed that symbioses involving two cooperating lineages can evolve under conditions where aggregate size, connectivity, and differentiation costs are high. This may explain why plants engage in symbiotic interactions with diazotrophic bacteria.

## Author Summary

The evolution of multicellularity is one of the most fascinating topics of evolutionary biology. Without multicellularity the incredible diversity of extant life would not be possible. In many multicellular organisms with specialized cells, some cell types become terminally differentiated (somatic cells) and lose the ability to reproduce new organisms while other cells maintain this ability (germline). Little is known about the conditions that favor the evolution of terminal differentiation in multicellular organisms. To understand this problem we have developed a computational model, inspired by multicellular cyanobacteria, in which the cells in an organism composed of two cell types (photosynthetic and nitrogen fixing) are allowed to evolve from germline to soma cells. We find three striking results. First, faster dividing cell types always evolve to become the germline. Second, the conditions under which we find different outcomes from the model are in good agreement with the different forms of development observed in multicellular cyanobacteria. Third, some conditions lead to a symbiotic state in which the two cell types separate into different lineages evolving independently of one another. Remarkably, cyanobacteria are also known to engage in symbiotic relationships with plants, producing fixed nitrogen for the plant in exchange for carbohydrates.

## Introduction

The reproduction and development of differentiated multicellular organisms follows a complex iterative pattern. Almost all differentiated multicellular organisms develop from a single pluripotent germ cell that divides and differentiates. Although terminally differentiated somatic cells contain all the necessary genetic information to produce whole organisms [1]–[3], they are unable to do so despite the potential cost in reproductive opportunities for the organism. In contrast, organisms composed of reversibly differentiated cells can reproduce through fragmentation or budding. Examples include most plants, and some animals such as corals, *hydra*, planarians, several echinoderms, and some annelid worms [4]–[7]. In these organisms, a fragment can regenerate the missing parts of the organism, resulting in several complete new organisms. During such regeneration, somatic cells in the fragments can sometimes de-differentiate and form a blastema (a group of undifferentiated cells) that regenerates the missing parts [6]. This means that somatic cells undergo reversible differentiation, and can revert back to their undifferentiated forms.

Multicellular cyanobacteria are some of the simplest multicellular organisms known. They are of particular interest because in some species, cells are terminally differentiated [8], [9], while in others, terminally differentiated cells have not been observed. Cyanobacteria have very diverse morphologies. They are found as single cells, multicellular filaments of undifferentiated cells, and differentiated multicellular filaments (with or without branching) [10]. In differentiated multicellular cyanobacteria, some cells specialise in photosynthesis while others specialise in nitrogen fixation. Only one genus of cyanobacteria (*Trichodesmium*) is known that could potentially exhibit reversible differentiation [11], [12]. In contrast, several terminally differentiating cyanobacteria are known, of which two examples are the genera *Anabaena* and *Nostoc*. These cyanobacteria are composed of two cell types: the vegetative cell (germline) and the heterocyst cell (soma). Vegetative cells are photosynthetic, reproduce through division, and are able to differentiate into heterocyst cells [13]. Heterocysts do not divide, have a thicker cell wall, and perform nitrogen fixation. They are also larger than vegetative cells. In this manner, vegetative cells obtain fixed nitrogen from heterocysts, and heterocysts obtain fixed carbon from the vegetative cells. These cyanobacteria have strongly regulated patterns of differentiation, forming heterocysts every 11 vegetative cells, with little variance in the number of vegetative cells between heterocysts [14]. Since the pattern of differentiation of cyanobacteria can not be explained solely through random differentiation [13], many studies have focused on understanding the mechanisms of pattern formation [13], [15]–[17]. Experimental evidence has identified three genes that play a key role in its regulation. NtcA, HetR and PatS all play a role in the differentiation mechanism of cyanobacteria. NtcA is a DNA binding factor that regulates the transcription of genes involved in nitrate transport and assimilation [18], HetR has been shown to be expressed shortly after heterocyst formation is induced when fixed nitrogen becomes scarce [19]. PatS is a gene that represses the formation of heterocysts and is believed to be produced by developing heterocysts and released to neighboring cells to prevent the formation of clusters of tightly spaced heterocysts [14].

A proximal explanation for the fact that heterocysts are terminally differentiated may be the physical constraints on cell division due to their thicker cell wall. However, the existence of the cyanobacterial genus *Trichodesmium*, in which cells perform nitrogen fixation and are capable of cell division [11], [12], suggests the possibility of other explanations.

The general question of why selection has favored the evolution of multicellularity and cell differentiation has been explored in many previous studies [20]–[28]. The evolution of multicellularity is faced with a similar conflict as the evolution of cooperation in social organisms. The conflict arises because natural selection favors the propagation of individual's with the highest fitness, while the evolution and maintenance of cooperation requires selection to favor individuals with a behavior that incurs them a cost in fitness while increasing the fitness of other individuals. In this scenario, individuals with a non-cooperating phenotype or cheaters would reap the benefits paying none of the costs and be therefore the most fit. Indeed, cooperation has been shown to only arise when the fitness cost to an individual is outweighed by the benefits conferred on related individuals, a concept explained by inclusive fitness theory [29]. Many forms of conflict mediation have been proposed to facilitate the maintenance of cooperation in multicellular organisms [20], [25], [26]. Unicellular bottlenecks and small propagule size are believed to be a main factor in the maintenance of cooperation by ensuring that new organisms are composed of highly related cells.

Once multicellularity evolves, cell differentiation and specialization can evolve, because it provides an increase in fitness to a group of related cells, which would not be otherwise possible [22], [26], [28]. Of all types of specialization, terminal differentiation, where cells lose their ability to reproduce new organisms may be the most extreme case of specialization.

Whether cells are reversibly or terminally differentiated, they must always cooperate if the organism is to survive. Given that cells must cooperate, why a specific cell type evolves to become the germline while others evolve to become the soma is a topic that has received little attention. One proposed explanation is that differentiation can autonomously arise as a result of stochastic chemical interactions within and between cells [30], and this can lead to terminal differentiation [31]. While this is a plausible hypothesis, it does not address the question of what evolutionary forces drive the evolution of different differentiation schemes such as reversible and terminal differentiation.

Using a spatially explicit approach, we model here the evolution of differentiation. Our model follows assumptions about multicellular cyanobacterial species, but is nonetheless sufficiently general to apply to other systems. We assume that the physiological interactions of cells with neighbouring cells affects their reproductive success. We find that the topology of interactions, the differentiation costs, and the relative division rate between different cell types can all play a role in the evolution of terminal or reversible differentiation. In addition, we find that some conditions can lead to the “speciation” of a multicellular organism into a symbiotic pair of organisms. In this case, the different cell types separate into two lineages evolving independently from each other. Our approach helps to identify some of the principal factors that led to the evolution of the diverse differentiation strategies seen in simple multicellular organisms, such as the cyanobacteria.

## Model

**Figure 1:**
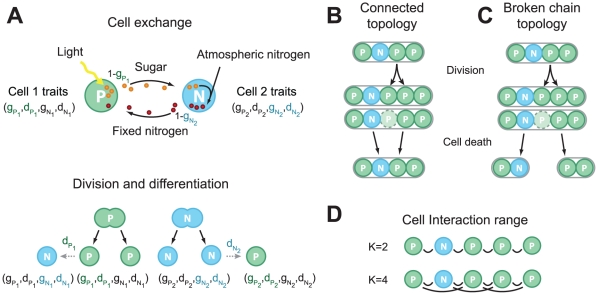
Model Illustration. (A) Cells have one of two phenotypes, photosynthetic 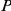 or nitrogen fixing 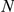. Every cell carries 4 traits 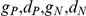 but only two traits influence a cell's behavior (shown in color). 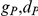 determine the behavior of photosynthetic cells and 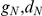 determine the behavior of nitrogen fixing cells. The case of two interacting cells is shown: photosynthetic cell 1 and nitrogen fixing cell 2. Cell 1 produces sugar through photosynthesis, keeping the fraction 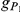 of the product for its own growth and division, the remainder, 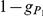, is given to cell 2. Cell 2 uses part of the sugar received to fix nitrogen, keeping the fraction 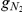 for its division and gives back 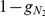 to cell 1. After a cell division, one of the daughter cells has a probability of differentiating according to the parent cell's differentiation rate and phenotype 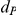 or 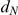. After a cell division, another cell is chosen at random for death regardless of its fitness. Two different filament topologies were investigated. (B) Connected topology, all cells remain connected after a cell death. (C) Broken chain topology, cell death results in the separation of its neighbours. (D) Effects of interaction range were investigated by increasing the number of connections between the cells and their nearest neighbours.

For this model we draw inspiration from the exchange of resources between cells in differentiated cyanobacteria. We consider a finite population of individuals or cells arranged in linear chains or filaments that exchange carbohydrates and fixed nitrogen with their neighbours (Fig. 1). Each cell can be of two types, either a photosynthetic cell or a nitrogen fixing cell which may produce only one resource, either carbohydrates or fixed nitrogen. Since cells are composed of both carbon and nitrogen, they need both elements for growth and division and therefore need to exchange these resources in order to reproduce.

The simulation can be divided in two phases, the resource production and exchange phase and the selection and evolution phase. In the first phase, the production and exchange of resources for every cell is calculated based on their traits and neighbors, and the fitnesses are computed. In the second phase, the evolution of the population proceeds in two steps. First, a cell is randomly selected for reproduction with a probability proportional to its fitness. Second, another cell is selected randomly for death, irrespective of its fitness.

### Cell traits

In the model, every cell is characterised by four evolvable traits (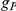, 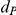, 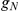, 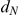) which may have any value in the range 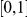 (Fig. 1A). Of these four traits, two traits (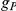, 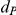) affect only photosynthetic cells, while the other two (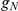, 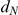) affect only nitrogen fixing cells. The traits 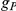 or 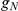 control how much of the resources produced by a cell are kept for its own growth and division, while the remaining fraction 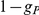 or 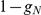 is given away to neighbouring cells. This means that a photosynthetic cell having a trait value of 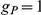 will keep all produced carbohydrates for its own cell growth while another cell with 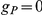 gives away all its produced carbohydrates. The traits 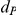 or 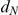 control the fraction of cells that differentiate into the other cell type immediately following a cell division. In other words, cells do not differentiate if they do not divide previously. For example, if a photosynthetic cell has the trait value 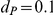, then 10% of its offspring cells will differentiate into nitrogen fixing cells. The individuals in our simulations evolve through mutation. This can occur every time a cell reproduces, at which time, traits in the daughter cell may mutate with probability 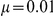, changing by a random amount that is uniformly distributed in the range 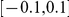.

### Cell fitness

Cell composition ratios of carbon to nitrogen (C∶N) have been estimated to be around 6∶1 for bacterioplankton [32]. Typical sugar molecules produced in photosynthesis contain 6 carbon atoms. Therefore we consider the biomass composition to be 1 unit of carbohydrates to 1 unit of fixed nitrogen. Assuming that this ratio remains constant in the cell, and therefore that cells require carbohydrates and fixed nitrogen in equal parts, their division rate will be limited by the least available resource.

Cell reproductive fitness is determined by division rate in the model. A cell's division rate depends on the amount of carbohydrates 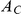 and fixed nitrogen 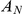 available for its reproduction. Given these considerations, we define the fitnesses 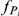 of a photosynthetic cell 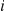 and 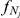 of a nitrogen fixing cell 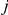 as
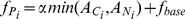
(1)

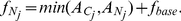
(2)Here 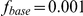 is a small constant that represents the base fitness and serves only to prevent the fitness from being zero, and 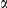 is a parameter that determines how fast a photosynthetic cell divides relative to a nitrogen fixing cell given the same amount of resources. Differences in cell division rate between cell types can result from differences in cell composition, cell size [33], the rate of biomass production, maintenance costs [34], and regulatory effects.

In the case of a nitrogen fixing cell, the total amount of carbohydrates 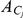 or fixed nitrogen 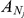 available to a cell for growth and division will be the fraction 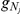 of received carbohydrates 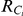 kept for reproduction minus the fraction 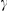 consumed in nitrogen fixation to supply the cell with fixed nitrogen for its own growth.
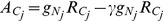
(3)

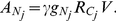
(4)Here 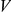 is the ratio that defines the amount of fixed nitrogen produced per carbohydrate consumed. The energetic costs of fixing nitrogen have been estimated to be 1 to 2 molecules of sugar for one molecule of ammonia [27], [35]. For simplicity, we have assumed 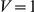.

To further simplify the model we assume that the nitrogen fixing cell is capable of regulating the amount of carbohydrates that it needs to consume for nitrogen fixation 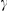 in order to achieve optimal growth. The optimal value of 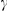 will depend on the ratio of carbohydrates to fixed nitrogen and should be 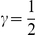 given the 1∶1 ratio assumed here. This leads to the following fitness function 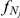 for the nitrogen fixing cells:
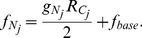
(5)


In the case of a photosynthetic cell, the amount of carbohydrates 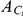 available for its growth will be the fraction 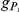 of produced carbohydrates 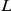 kept for growth. The amount of fixed nitrogen 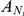 available will be equal to the amount of fixed nitrogen received from neighboring nitrogen fixing cells:
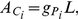
(6)

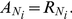
(7)Here we assume that all photosynthetic cells produce one unit of carbohydrates 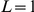. This leads to the following fitness function 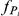 for the photosyntetic cells:
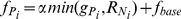
(8)


The amount of resources received from other cells will depend on many factors, such as the cell interaction topology, the interaction range, and the traits of the other cells (Fig. 1). 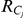 is the amount of sugar received by nitrogen fixing cell 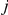, where
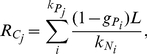
(9)

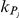
 is the number of photosynthetic cells interacting with cell 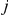 and 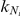 is the number of nitrogen fixing cells interacting with cell 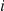. Meanwhile, 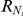 is the amount of fixed nitrogen received by photosynthetic cell 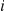 from interacting nitrogen fixing cells, where
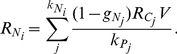
(10)The range of values 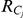 and 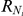 can have will depend on the interaction range, the type and trait values of neighboring cells. For example, when K = 4, a nitrogen fixing cell could receive at most 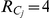 units of carbohydrates if it had 4 neighboring photosynthetic cells that gave away all their carbohydrates.

To study the effects of differentiation costs we have modeled them as a reduction in the fitness of a differentiated cell by a fraction 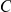, such that the fitness of the cell becomes 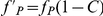. After the first time a cell is chosen for division, this cost is removed. A differentiation cost modeled this way is equivalent to a reduction in resources available for growth by a proportional amount. Using a constant amount instead of a factor does not qualitatively change the results, as is shown in the results. Differentiation costs are expected to exist in differentiating cells because differentiation requires a cell to degrade the proteins expressed in its previous cell type. The degradation of these proteins therefore incurs a cost of energy or materials. It is also known to incur costs in higher organisms [36].

Fitness in our model is translated into a proportional probability that a cell will be chosen for reproduction every iteration. This probability of division is given by 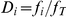, where 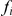 is the fitness of cell 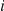 and 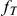 is the sum over all fitness values of the cells in the population.

### Topology of cell exchange

In this model, cells are arranged in linear chains. When a cell reproduces, a new cell with the same traits is inserted in the chain between its parent and a neighbour (Fig. 1B,C). We investigate two filament topologies that result as a consequence of the type of cell death considered. In the connected topology (Fig. 1B), a cell chosen for death is simply removed from the chain, with one of the neighbours taking the place of the removed cell. In the broken chain topology (Fig. 1C), the chain is broken in two parts when a cell chosen for death is removed, hence separating some of the neighbours of the removed cell. In addition, we study the effects of varying the distances at which fixed nitrogen and fixed carbon are exchanged by changing the interaction range 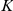 between cells (Fig. 1D). The interaction range represents the distance that nutrients are allowed to diffuse between cells due to proximity or through the transport of nutrients by vascular systems. Here we have considered the use of constant interaction strengths between cells, and vary instead only the number of neighboring cells that a cell can reach or interact with. We also investigate the use of an interaction strength defined by a Gaussian function which is presented in the supplementary information.

### Developmental strategies

**Figure 2:**
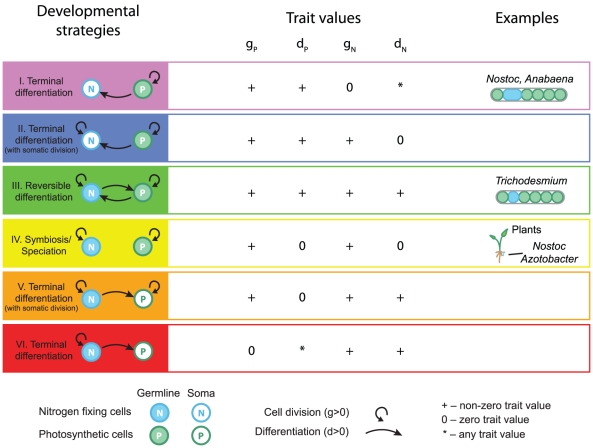
Possible developmental strategies. Developmental strategies classified based on the trait averages (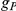, 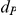, 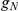, 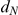). The arrows that point from one cell type to itself represent investment in growth and division (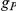 or 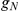) while the arrows between cell types represent differentiation (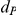 or 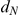). Six possible developmental strategies exist: I. terminal differentiation with photosynthetic germline and non-dividing nitrogen fixing soma (violet), II. terminal differentiation with photosynthetic germline and dividing nitrogen fixing soma (blue), III. Reversible differentiation (green), IV. symbiosis (yellow), V. terminal differentiation with nitrogen fixing germline and dividing photosynthetic soma (orange), and VI. terminal differentiation with nitrogen fixing germline and non-dividing photosynthetic soma (red). The sign (+) indicates that the trait value is greater than zero. The asterisk (*) indicates that the trait may have any value. For the purposes of classification, we considered trait values bellow the threshold of 0.05 to be effectively 0. On the right, the different developmental strategies are shown to represent observed developmental strategies in cyanobacteria. I. terminal differentiation is seen in heterocystous cyanobacteria in *Nostoc* and *Anabaena*, III. reversible differentiation is observed in the cyanobacterium *Trichodesmium*, and symbiosis is observed between diverse plants and the cyanobacterium *Nostoc*.

The four traits (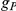, 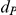, 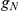, 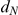) can evolve through mutation and selection to arrive at different sets of values. For the population to be viable both nitrogen fixing cells and photosynthetic cells must exist and exchange resources. This restriction implies that some sets of values such as (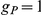, 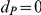, 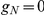) can never evolve because no nitrogen fixing cells would be produced in a homogenous population of cells with this genotype. Fig. 2 provides a classification for the 6 genotypes that can evolve, which we will refer to as developmental strategies. Two developmental strategies correspond to terminally differentiating genotypes, where the nitrogen fixing cell is terminally differentiated (I, violet), or where the photosynthetic cell is terminally differentiated (VI, red). Two strategies are intermediate cases of terminal differentiation, where differentiated cells still divide (II, blue; V, orange). One strategy corresponds to reversible differentiation where both cells can differentiate into the other cell type (III, green). The last strategy corresponds to symbiosis, where both cells reproduce but do not differentiate (IV, yellow). With this approach, a photosynthetic cell in the model can evolve from germline to soma, if the conditions imposed in the model favor that transition through mutation and selection. In this manner, we investigate the conditions that favor the evolution of the different developmental strategies.

## Results

### Evolutionary stability of developmental strategies

**Figure 3:**
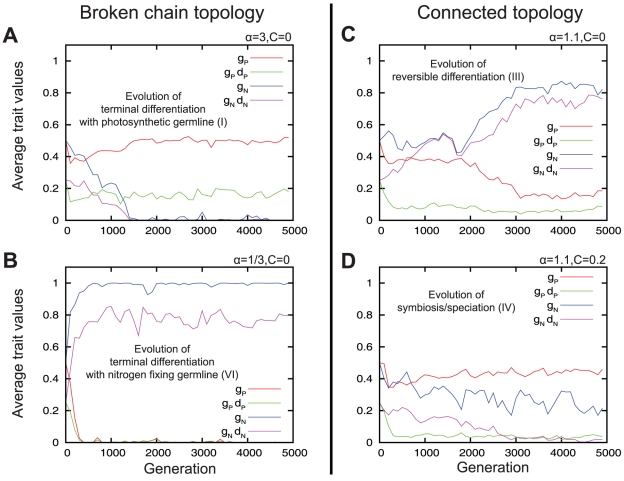
Examples of the evolution of the population trait averages. Evolution of trait averages (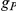, 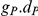, 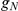, 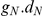) of 400 cells over 5000 generations under different conditions of relative division rate 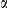, filament topology, and differentiation costs 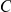. (A,B) simulations of the broken chain topology differing only in the relative division rates 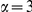 and 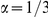, respectively. (B,D) simulations of the connected topology differing only in the differentiation cost 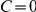 and 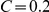, respectively. All simulations shown here have interaction range set to 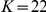.

We analysed the evolution of the variable traits (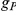, 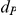, 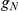, 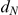) in populations of 400 cells starting with the set of initial trait values (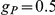, 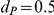, 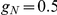, 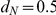). At the start of the simulation, all photosynthetic and nitrogen fixing cells are homogeneous with respect to their traits. Cells were initially placed in a single filament with periodic boundary conditions and randomly assigned as photosynthetic or nitrogen fixing with equal probability. The four panels in Fig. 3 show examples of the evolution of the population average of each trait in four different conditions. Each generation corresponds to 400 cell deaths and divisions. Instead of the differentiation rates 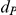 and 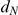, the products 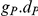 and 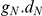 are plotted, because these express the effective rate of differentiation after cell division. For all simulations in Fig. 3, it can be seen that the average trait values (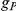, 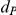, 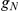, 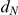) evolve rapidly to a point were they begin fluctuating around a state which depends on the parameters of the simulation. The parameters investigated are the relative division rate 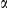, differentiation cost 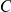, filament topology, and interaction range 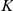. Using the averages of the variable traits we classify the evolved developmental strategy of the population according to Fig. 2. For the purpose of classification, we consider trait values below the threshold of 0.05 to be effectively 0. Fig. 3A shows the evolution of the averages of variable traits (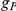, 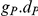, 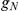, 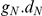) over 5000 generation in an evolving population using a broken chain topology, where photosynthetic cells have a relative division rate three times faster (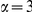) than nitrogen fixing cells, and with no differentiation costs (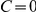). We can see that in the final generation, photosynthetic cells keep half of the produced carbohydrates (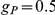) for their own cell growth and division, and differentiate at a rate of (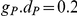), while the nitrogen fixing cells do not keep any fixed nitrogen (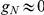) and therefore do not divide nor differentiate (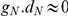). Using Fig. 1 we classify this strategy as terminal differentiation with a photosynthetic germline (I). Fig. 3B shows a simulation in the same conditions as in Fig. 3A, except that the photosynthetic cells divide three times more slowly (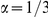). In this case we observe that the final strategy is terminal differentiation with a nitrogen fixing germline (VI). Figs. 3C and 2D show simulations in the connected topology with slightly faster dividing photosynthetic cells (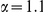). In Fig. 3C there are no differentiation costs (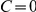) and the final strategy corresponds to reversible differentiation (III). In Fig. 3D there is a differentiation cost (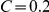) and the final strategy corresponds to the case of symbiosis (IV) (the different cell types evolve into separate lineages).

### Evolved developmental strategies are insensitive to initial conditions

**Figure 4:**
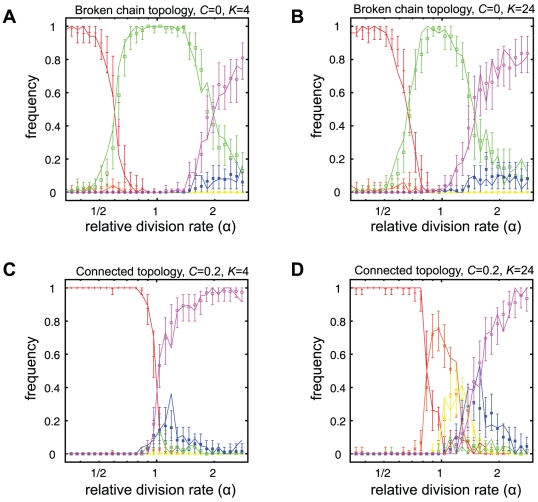
Frequency of evolved developmental strategies. The solid lines show the frequency of evolution of each strategy for varying relative division rates 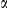 (50 simulations per 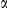 value). Data points and error bars show the average and 95% confidence interval for simulations using random initial conditions. Confidence intervals were calculated using the bootstrap method on 500 simulations per 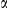 value. Each strategy is represented by a different colour according to Fig. 2. Two different cases are shown: (A,B) broken chain topology with no differentiation costs 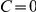, (C,D) connected topology with differentiation costs 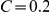. Each case is shown for two different interaction ranges 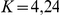 corresponding to the panels on the left, and right, respectively. Each simulation was performed with 400 cells over 10 000 generations. Relative division rates (x-axis) are in logscale.

Next we investigate the sensitivity of the evolved developmental strategy to the initial traits (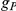, 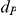, 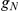, 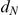) and whether different developmental strategies may evolve in the same conditions. In Fig. 4, the solid lines show the plots of frequencies of the evolution of each developmental strategy when 50 stochastic simulations are carried out starting the simulation from a homogeneous population with initial traits (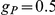, 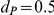, 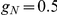, 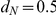). In contrast, based on random initial conditions, the data points and error bars in Fig. 4 show the average and 95% confidence intervals for the frequency of evolved developmental strategies, respectively. This is estimated using bootstrapping from 500 simulations with random initial traits (see supplementary information for details). Each plot shows how the frequencies change with varying relative division rate. The panels on the top (Fig. 4A,B) show the results in the case of the broken chain topology with no differentiation costs (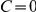). Figs. 4C,D show the case of the connected topology with differentiation costs (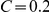). Simulations for two different cell interaction ranges (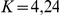) are shown in Fig. 4. Other parameter combinations are shown in Fig. S1 and discussed in Text S1. Different mutation rates (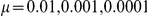) and population sizes (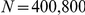) were tested and found to only change the number of generations needed for the system to evolve to the final developmental strategy. Lower mutation rates and larger population sizes required more generations for the population to reach the equilibrium compared to higher mutation rates or smaller population sizes.

The confidence intervals observed in Fig. 4 are narrow, indicating that the developmental strategies which evolve are rather insensitive to the trait values of the initial population. Only a single strategy is generally seen to evolve under a set of conditions. However, at the points where a transition is observed between the most frequent strategies, two or more strategies evolve at appreciable frequencies, and that coincide with broader confidence intervals. For example, at 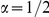 in Fig. 4A (broken chain topology, 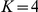), a transition in the most frequently evolved strategy can be seen between terminal differentiation with nitrogen fixing germline (VI, red) and reversible differentiation (III, green). At 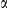 slightly larger than 1 in Fig. 4D (connected topology, 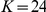), many strategies can be seen to evolve with some frequency.

At large differences in division rates (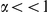 or 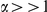), when one cell divides much faster than the other, terminal differentiation without somatic division (I, violet and VI, red) evolves. Furthermore, it is the faster dividing cell type that becomes the germline. Hence, at low relative division rates (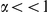), when nitrogen fixing cells are dividing faster, terminal differentiation with a nitrogen fixing germline (VI, red) is the most frequently evolved strategy. Conversely, at high relative division rates (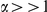), when photosynthetic cells are the more rapidly dividing cells, terminal differentiation with a photosynthetic germline (I, violet) is the most frequently evolved strategy.

### Cell types with higher division rates evolve to become the germline

**Figure 5:**
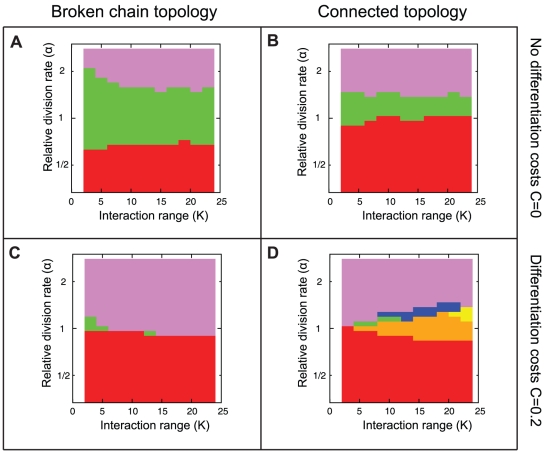
All possible developmental strategies evolve under some sets of conditions. The panels show the most frequently evolved developmental strategies depending on the cell interaction range 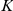 and the relative division rate of photosynthetic cells 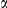. (A,C) show the results simulated in the broken chain topology with no differentiation costs 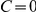 and with differentiation costs 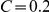, respectively. (B,D) show the results in the connected topology with the same two differentiation costs. Simulations were repeated 50 times for each parameter combination, and the population size was 400. The color represents the strategy found to evolve most frequently, with color codes as in Fig. 2. Relative division rates (y-axis) are in logscale.

To further examine the conditions which determine the most frequently evolved developmental strategies, we performed simulations for different relative division rates ranging from 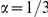 to 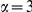, interaction ranges ranging from 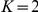 to 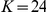, two different filament topologies (broken chain and connected), and two values of differentiation costs (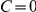 and 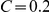). Fig. 5 shows the most frequently evolved strategies (represented as colours classified in Fig. 5A) for each combination of parameters 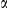 and 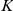 simulated 50 times starting with initial traits (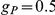, 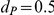, 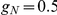, 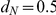). All cases confirm that terminal differentiation (I, violet and VI, red) evolves at the extremes of relative division rate, in which the fastest dividing cell becomes the germline.

In the broken chain topology, both with no differentiation costs 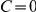 (Fig. 5A) and with differentiation costs 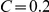 (Fig. 5C), only three developmental strategies evolve. These are differentiation with a photosynthetic germline (I, violet), reversible differentiation (III, green), and terminal differentiation with a nitrogen fixing germline (VI, red). In both cases it can be seen that the main factor influencing the evolved developmental strategy is the relative division rate (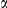), with little dependency on the interaction range of the cells (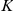). In Fig. 5A, where no differentiation costs were included, fast dividing photosynthetic cells (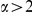) result in the evolution of terminal differentiation with photosynthetic cells as the germline (I, violet). Slow dividing photosynthetic cells (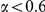) also lead to the evolution of terminal differentiation, but in this case the nitrogen fixing cells become the germline (VI, red). For intermediate relative division rates (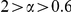), reversible differentiation (III, green) is the evolved strategy.

When a differentiation cost 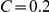 is considered (Fig. 5C), the range under which reversible differentiation (III, green) evolves is limited to 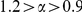 at low 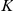 values. Conversely, the range of 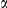 values under which terminal differentiation (I, violet and VI, red) evolves increases.

For the connected topology with no differentiation costs 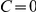 (Fig. 5B), the result is qualitatively similar to the one observed for the broken chain topology with 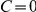 (Fig. 5A). In both cases only three strategies are observed to evolve most frequently, the two types of terminal differentiation without somatic division (I, violet and VI, red) and reversible differentiation (III, green).

### All developmental strategies evolve frequently in some conditions

Remarkably, with differentiation costs and a connected topology (Fig. 5D), all developmental strategies evolve in some range of conditions. Reversible differentiation (III, green) is reduced to a very narrow range of conditions with intermediate values of interaction ranges (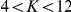) and slightly faster dividing photosynthetic cells (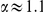). When 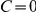, the range of conditions previously occupied by reversible differentiation (III, green) is replaced by terminal differentiation with somatic division (II, blue and V, orange) at shorter interaction ranges (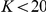), and symbiosis (IV, yellow) at longer interaction ranges (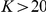).

### Model modifications

One assumption we have made that may not apply to other systems is that nitrogen fixing cells are only able to fix nitrogen if they obtain carbohydrates from photosynthetic cells. This results in an asymmetry in the model because photosynthetic cells do not require fixed nitrogen to perform photosynthesis, though they require it for cell growth and division. We show in Fig. S4 that the results presented here do not qualitatively change when we modify the model to enable nitrogen fixing cells to fix nitrogen independently of the carbohydrates received.

We also explored other modifications and found that in all cases the results have remained qualitatively the same. In Fig. S5 we show the results of using a constant differentiation cost instead of a cost that decreases the resources available to a cell by a fraction. In Fig. S6, we show the results when using a Gaussian function to define the interaction strengths between cells.

## Discussion

### Importance of differences in division rates

The results shown here establish a strong link between the relative division rate of different cell types and the cell type that becomes the germline in a multicellular organism. Figs. 4 and 5 indicate that when one cell type divides faster than the other, it evolves to become the germline. This result is found to be independent of the differentiation costs (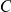), filament topology, and interaction range (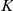). The reason can be explained intuitively by noting that an organism that requires both cell types will divide faster when the fastest dividing cell type is the one that produces the other cell type as needed. Hence the faster dividing cell types are the ones which remain pluripotent. For example this pattern is seen in plants, where cells in the apical meristems generating shoots and roots consist of rapidly dividing undifferentiated cells [37], [38]. Equivalently, one can interpret this as a situation in which cells that have a higher fitness at the individual level are the ones that become the germline.

When division rates of the different cell types are comparable and 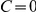, our model shows that reversible differentiation (III, green) evolves (Figs. 5A and 5B). This corresponds to the case of differentiated cells that have the ability to de-differentiate into another cell type. Examples exist in many plants and in some animals capable of regeneration [5], [6]. Although terminal differentiation is found to evolve in the widest range of conditions, reversible differentiation can evolve in conditions where the division rates of different cell types are comparable. The latter can happen even in the absence of selection for the ability to regenerate or reproduce by fragmentation (Figs. 5B,D).

It is important to note that large differences in cellular division rates are a necessary but insufficient condition for a cell type to become the germline. The fast growth rate of a cell type must not harm the fitness of the organism as a whole, otherwise faster growing cells such as cancer cells would become the germline more often. Such an eventuality has occurred only on rare occasions [39], [40].

### Role of filament topology and interaction range

Cell interaction affects developmental strategies in two ways. First, the broken chain topology (Fig. 5A) increases the range of conditions under which reversible differentiation (III, green) evolves when compared to the connected topology (Fig. 5B). The reason can be understood if we consider that reversible differentiation increases the survival of filaments in response to fragmentation. By ensuring that either cell type can produce the other cell type, the probability that a fragment will carry only non-differentiating cells is reduced. A similar argument can be made to explain why symbiosis (IV, yellow) does not evolve in the broken chain topologies under any conditions (Figs. 5A and 5C). In these topologies, broken fragments never come into contact again, meaning that once a symbiotic pair within a filament is split, it will be condemned to death. Hence, such mutants can never become fixed in the population.

The effect of interaction range (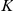) is mainly seen in connected topologies. In this case, all possible developmental strategies evolve in at least one set of conditions (Fig. 5B,D). For example, the symbiotic state (IV, yellow) that was not found in broken chain topologies, occurs in the connected topology if interaction ranges (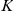) are sufficiently high (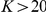) and if there are differentiation costs (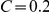). In the case with differentiation costs, increasing the interaction range leads to a decrease in the range of relative division rates under which terminal differentiation evolves, while the range for other strategies expands (Fig. 5D). Higher interaction ranges (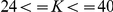) in the connected topology are shown in Fig. S3 and discussed in Text S1. They lead to a slight increase in the range of relative division rates in which symbiosis (IV, yellow) and terminal differentiation with a nitrogen fixing germline and somatic division (V, orange) occur.

It is well known that topologies with few interactions promote cooperative behaviour, while fully connected topologies, where all individuals interact with each other, result in the invasion of cheaters [41], [42]. This has already been shown to be the case in a model of cyanobacteria [27], in which populations of vegetative and heterocyst cells are driven to extinction in the fully connected case. Here, we have analysed topologies that are far from the fully connected case, and where several forms of cooperation are stable. By varying the relative division rate, several developmental strategies such as reversible differentiation and symbiosis can evolve in the same filament topology and interaction range (Fig. 5). These developmental strategies are neither altruistic nor selfish, since both cell types can divide. Hence, the mapping of our present results to established concepts in social biology may require further work.

### Correspondence to developmental strategies in cyanobacteria

Multicellular cyanobacteria have evolved several of the developmental strategies seen in this model. Terminally differentiating cyanobacteria such as *Anabaena* or *Nostoc* have filamentous forms composed of two different cell types: vegetative cells that are photosynthetic, divide and differentiate into the other cell type, and heterocyst cells that fix nitrogen and are unable to divide. The latter can be distinguished by their larger size and thicker cell walls [8]. Our model provides clues to why heterocystous cyanobacteria form terminally differentiated heterocysts that do not divide. An ad-hoc explanation based on a proximal cause is that a heterocyst's thicker cell wall impedes it from undergoing cell division. However, our results provide an alternative explanation. In light of the model, a thicker cell wall corresponds to added costs and therefore a slower division rate. Under this condition, the developmental strategy that maximises the organism's fitness is terminal differentiation without somatic division (I, violet and VI, red) (Fig. 5C). This means that the ultimate reason why heterocysts do not divide is not necessarily due to mechanistic constraints, but rather a result of evolutionary constraints.

The only known example of potentially reversibly differentiated cyanobacteria is *Trichodesmium*. In species of this genus, different cell types are morphologically indistinguishable. However, differences at the level of expression of nitrogenase exist, and nitrogen fixation is shown to occur in distinct cells found across the filaments [11]. Although cells are differentiated in their expressed protein and function, both cell types maintain their ability to divide [43], [44]. While no direct experiment has shown that cells in *Trichodesmium* reversibly differentiate, the fact that the fraction of nitrogen fixing cells varies with daily rhythmicity, reaching a maximum of 24% during the day and a minimum of 5% before dawn, suggests that the nitrogen fixing cells reversibly differentiate into photosynthetic cells [45]. In this case again, our results provide some insights as to why cells that are specialised in nitrogen fixation (therefore similar to heterocysts) are not terminally differentiated, but are still capable of dividing and of reverting back to a photosynthetic phenotype. Since both cell types are structurally similar, they can be expected to have similar division rates. The results shown in Figs. 4A and 5A predict that reversible differentiation (III, green) should be the most frequently evolved developmental strategy in this case.

So far, no known examples of multicellular cyanobacteria exist in which terminally differentiating nitrogen fixing cells (heterocysts) are capable of cell division (II, blue). While this can simply reflect our incomplete knowledge, our results suggest that such developmental strategies are evolutionarily unstable (Fig. 5A–D).

### Symbiosis/speciation

The finding that symbiosis evolves in a connected topology under several conditions of relative cell division rate and differentiation costs points to some interesting evolutionary possibilities. One is that some organisms may have speciated as a result of changing conditions that initially selected for terminal or reversible differentiation, but later changed to favour a symbiotic state. Potential support for this idea comes from a recently sequenced cyanobacterium named UCYN-A that is closely related to a member species of the genus *Cyanothece* [46]. *Cyanothece* are unicellular circadian cyanobacteria capable of photosynthesis and nitrogen fixation by temporally separating the two processes. The newly sequenced relative of *Cyanothece* lacks the genes necessary to perform photosynthesis found in *Cyanothece* species [46]. Instead, it has only the genes necessary for nitrogen fixation. Because it is unable to perform photosynthesis, it is dependent on obtaining its carbohydrates from the environment or from other organisms. This suggests that a scenario in which cyanobacteria speciate into symbiotic or interacting collectives is possible. In effect, chloroplasts, which are endosymbionts that descended from cyanobacteria, are a likely endpoint of such a scenario. In this case, chloroplasts provide the host plant with fixed carbon while the plant is the intermediary that provides fixed nitrogen.

Plants have never evolved the ability to fix nitrogen. They absorb it from the environment or rely instead on symbiotic diazotrophic bacteria such as the cyanobacterium *Nostoc* to fix nitrogen in exchange for carbohydrates produced by the photosynthetic plant [13]. The vascular system of plants conceptually changes the topology of cell interactions from a chain to a connected topology with high interaction ranges, allowing photosynthetic plant cells to exchange nutrients with the nitrogen fixing cyanobacteria in the roots of the plant. Our results show that in such conditions (Figs. 4D and 5D), a symbiotic relationship (IV, yellow) where the nitrogen fixing cells evolve independently from the photosynthetic cells is a frequently evolved strategy. The range of 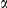 values in which symbiosis evolves is seen to increase with higher differentiation costs (Fig. S2 and Text S1) and interaction ranges (Fig. S3 and Text S1). These results suggest that the symbiotic relationship between plants and cyanobacteria may be evolutionarily more stable than the alternative scenario, in which plants would fix their own nitrogen.

### Generality of the model

While this model draws inspiration from differentiated cyanobacteria, the results found here may apply to a wider range of biological systems. In essence, the model describes the evolution of a simple multicellular organism or population with two types of individuals that produce different resources, but require both resources to reproduce. Hence, these individuals need to interact and exchange resources. By considering the exchange of fitness benefits as a form of resource exchange, a cell type in an organism that serves a structural function can also be analysed using such a model. In the supplementary information (Text S1) we present the results of several modifications to the model which do not qualitatively change the results found. The modifications we considered comprise a nitrogen fixing cells that do not need carbohydrates to fix nitrogen (Fig. S4), a fixed differentiation cost instead of a fractional cost (Fig. S5), and a Gaussian function to describe the interaction strengths (Fig. S6). In all cases we found that faster dividing cells evolve to become the germline, and all developmental strategies can evolve in some range of conditions. These results lend support to the idea that the observations we made do not just apply to cyanobacteria but can apply to a range of other simple differentiated multicellular organisms.

### Conclusion

This model shows that in simple organisms, the optimum developmental strategy depends on how cells divide and interact. We have shown that the topology of interactions, the interaction range, the differentiation costs, and the relative division rate between cell types all play a role in the type of differentiation that evolves. However, the difference in cell division rates is the main factor determining the type of differentiation that evolves. Furthermore, it determines the cell type that becomes the germline. Hence, we establish for the first time the conditions that drive the evolution of terminal and reversible differentiation.

## Supporting Information

Figure S1
**Frequency of evolved developmental strategies.** The plots show the frequency of evolution of each strategy with varying relative division rates 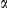 (50 simulations per 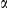 value). Each strategy is represented by a different color according to the key on the bottom. Four different cases are shown: (row A) broken chain topology with no differentiation costs (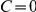), (row B) broken chain topology with differentiation costs (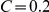), (row C) connected topology with no differentiation costs (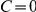), and (row D) connected topology with differentiation costs (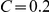). The plots in the three different columns correspond to different interaction ranges (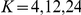), as shown above each column. Simulations were performed with 400 cells over 5000 generations.(PDF)

Figure S2
**Most evolved developmental strategies in the connected topology with higher differentiation costs.** The simulations were performed with varying cell interaction range 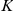 and photosynthetic cell relative division rate 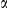 with differentiation cost (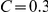). Simulations were repeated 50 times for each parameter combination and the population size was 400. The color represents the most frequently evolved strategy coded according to Figure 2 in the main text.(PDF)

Figure S3
**Most evolved developmental strategies in the connected topology with high interaction ranges.** The simulations were performed with cell interaction range 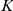 between (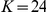) and (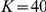). The two panels show the results of the simulations (A) with no differentiation costs (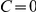) and (B) with differentiation costs (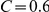). Simulations were repeated 50 times for each parameter combination, and the population size was 400. The color represents the most frequently evolved strategy coded according to Figure 2 in the main text.(PDF)

Figure S4
**Most evolved developmental strategies in simulations where different cell types have symmetric fitnesses.** Panels (a) and (c) show the results of the broken chain topology. Panels (b) and (d) show the results in the connected chain topology. The simulations were performed with varying cell interaction ranges 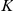 and photosynthetic cell relative division rates 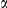, (a,b) with no differentiation costs (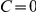) and (c,d) with differentiation costs (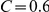). Simulations were repeated 50 times for each parameter combination, with population sizes of 400. The color represents the most frequently evolved strategy coded according to Figure 4 in the main text.(PDF)

Figure S5
**Model modification with a constant differentiation cost.** Frequency of evolved developmental strategies using a constant differentiation cost 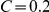 in the connected chain topology. The plots show the frequency of evolution of each strategy with varying relative division rates 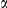 (30 simulations per 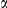 value). Each strategy is represented by a different colour according to the color key in Figure 2. The plots in the three different columns correspond to different interaction ranges (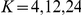), as shown above each column. Simulations were performed with 200 cells over 5000 generations.(PDF)

Figure S6
**Model modification with a Gaussian function for interaction strength.** Frequency of evolved developmental strategies using an interaction strength defined by a gaussian function 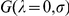 with varying standard deviation 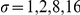 in the connected chain topology. The plots show the frequency of evolution of each strategy with varying relative division rates 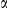 (30 simulations per 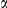 value). Each strategy is represented by a different color according to the color key in Figure 2. Simulations were performed with 200 cells over 5000 generations.(PDF)

Figure S7
**Comparison of mean and median of population trait values.** Evolution of population trait means and medians (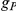, 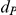, 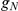, 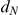) of 200 cells over 5000 generations in the broken chain topology, with relative division rate 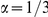 and interaction range 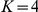.(PDF)

Text S1 **Other model results, modifications and method details.**
(PDF)
