# Correction: Differences in Cell Division Rates Drive the Evolution of Terminal Differentiation in Microbes

**DOI:** 10.1371/annotation/cea2d6e3-28ce-4284-84d1-08e9453e773e

**Published:** 2012-05-17

**Authors:** João F. Matias Rodrigues, Daniel J. Rankin, Valentina Rossetti, Andreas Wagner, Homayoun C. Bagheri

The affiliations for the third and fifth author were incorrect. The third Author, Valentina Rossetti, is not affiliated with #2 but with #1 Institute of Evolutionary Biology and Environmental Studies, University of Zurich, Zurich, Switzerland. The fifth author, Homayoun C Bagheri, is not affiliated with #2 but with #1 Institute of Evolutionary Biology and Environmental Studies, University of Zurich, Zurich, Switzerland. 

