# Supplementary material for: Differences in Cell Division Rates Drive the Evolution of Terminal Differentiation in Microbes
Source: PLoS Comput Biol. 2012 Apr 12;8(4):e1002468. doi: 10.1371/journal.pcbi.1002468 (PMC3325182; doi:10.1371/journal.pcbi.1002468)
Supplement: Figure S1 — Frequency of evolved developmental strategies. The plots show the frequency of evolution of each strategy with varying relative division rates (50 simulations per value). Each strategy is represented by a different color according to the key on the bottom. Four different cases are shown: (row A) broken chain topology with no differentiation costs (), (row B) broken chain topology with differentiation costs (), (row C) connected topology with no differentiation costs (), and (row D) connected topology with differentiation costs (). The plots in the three different columns correspond to different interaction ranges (), as shown above each column. Simulations were performed with 400 cells over 5000 generations. (PDF) [file pcbi.1002468.s001.pdf]

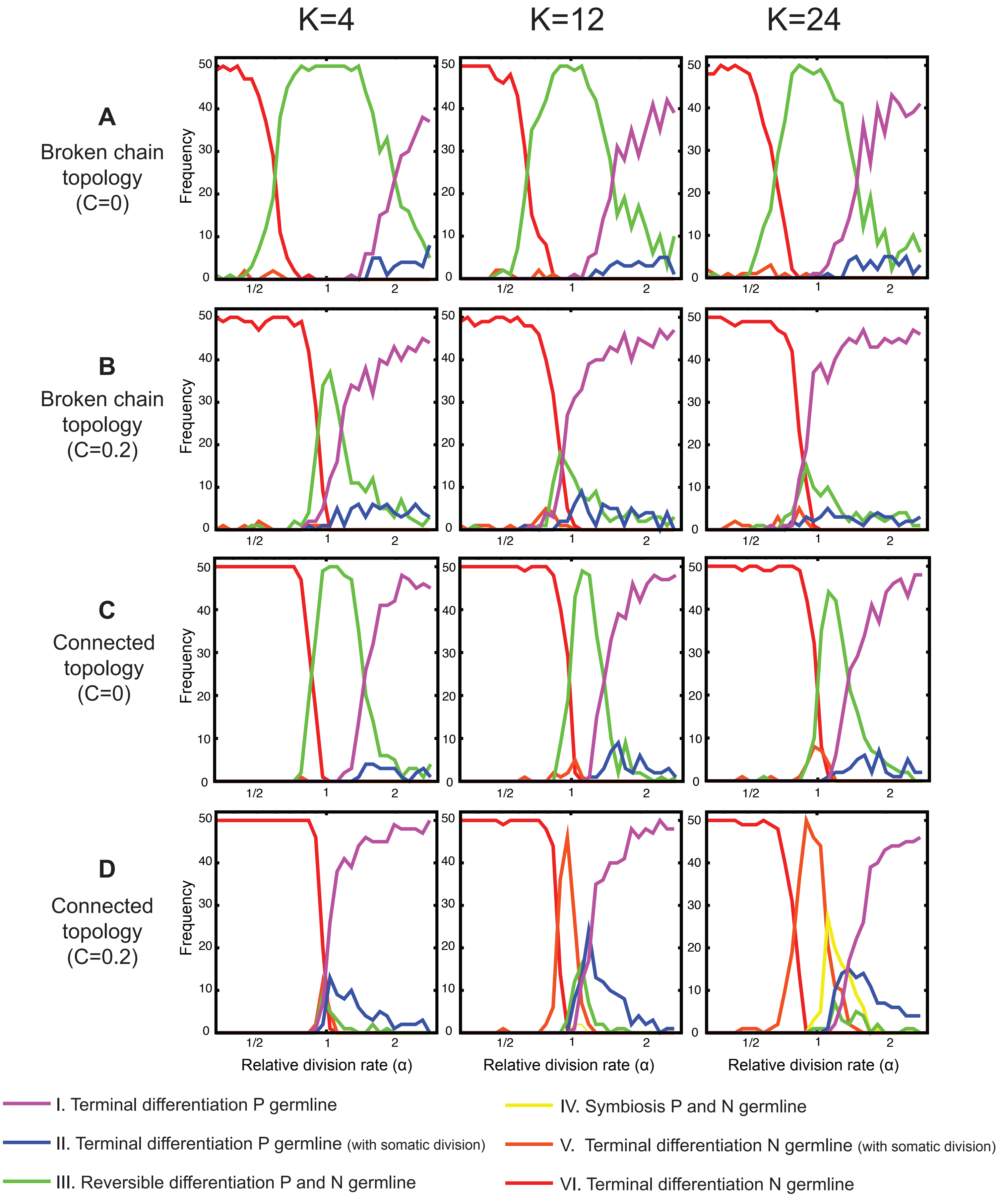

K=4
K=12
K=24
A
Broken chain topology (C=0)
B
Broken chain topology (C=0.2)
C
Connected topology (C=0)
D
Connected topology (C=0.2)
Frequency
0
10
20
30
40
50
1/2
1
2
Relative division rate (α)
I. Terminal differentiation P germline
II. Terminal differentiation P germline (with somatic division)
III. Reversible differentiation P and N germline
IV. Symbiosis P and N germline
V. Terminal differentiation N germline (with somatic division)
VI. Terminal differentiation N germline
