# Supplementary figures and images for: Differences in Cell Division Rates Drive the Evolution of Terminal Differentiation in Microbes
Source: PLoS Comput Biol. 2012 Apr 12;8(4):e1002468. doi: 10.1371/journal.pcbi.1002468 (PMC3325182; doi:10.1371/journal.pcbi.1002468)

Relative division rate ( $\alpha$ )

2  
1  
 $1/2$

0

5

10

15

20

25

Interaction range (K)

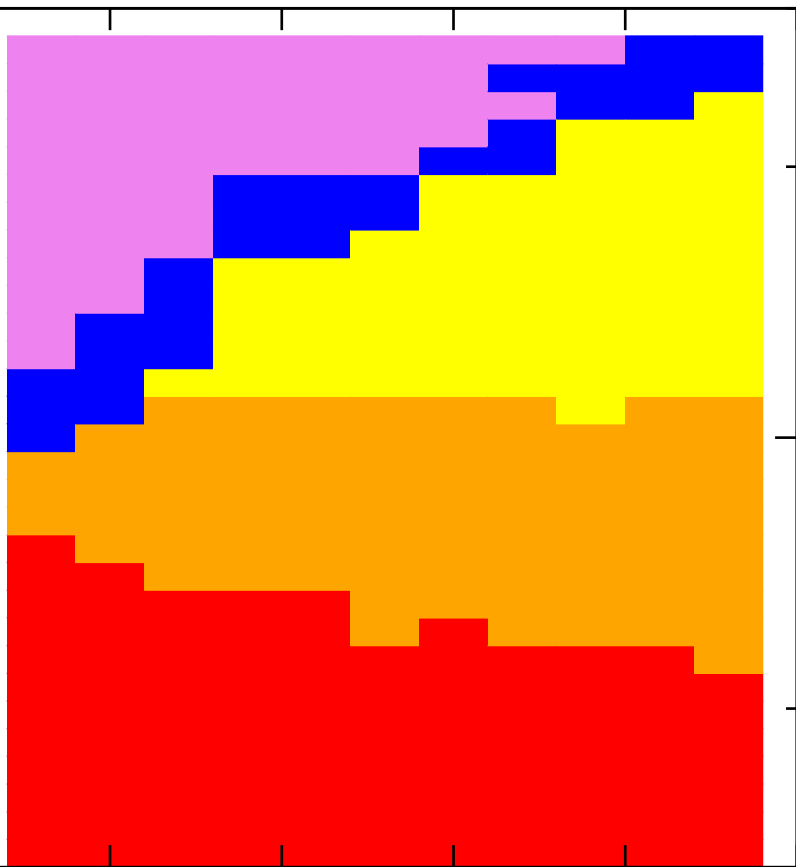

Supplement: Figure S2 — Most evolved developmental strategies in the connected topology with higher differentiation costs. The simulations were performed with varying cell interaction range and photosynthetic cell relative division rate with differentiation cost (). Simulations were repeated 50 times for each parameter combination and the population size was 400. The color represents the most frequently evolved strategy coded according to Figure 2 in the main text. (PDF) [file pcbi.1002468.s002.pdf]

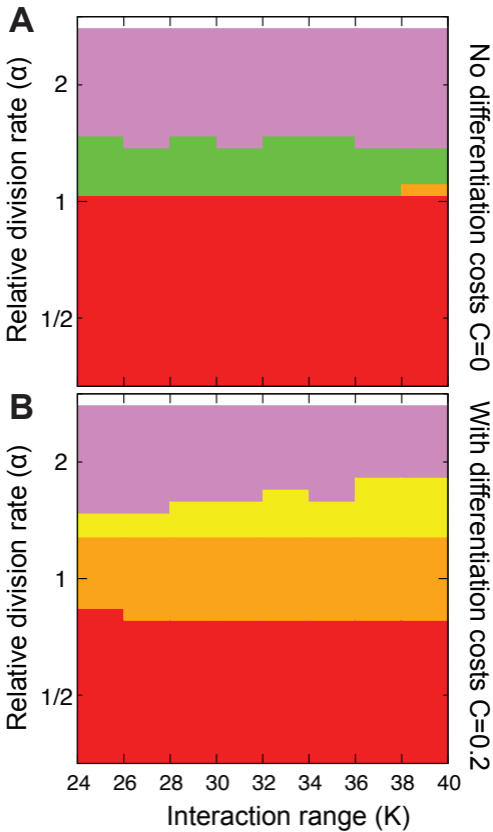

Supplement: Figure S3 — Most evolved developmental strategies in the connected topology with high interaction ranges. The simulations were performed with cell interaction range between () and (). The two panels show the results of the simulations (A) with no differentiation costs () and (B) with differentiation costs (). Simulations were repeated 50 times for each parameter combination, and the population size was 400. The color represents the most frequently evolved strategy coded according to Figure 2 in the main text. (PDF) [file pcbi.1002468.s003.pdf]

Broken chain topology

Connected topology

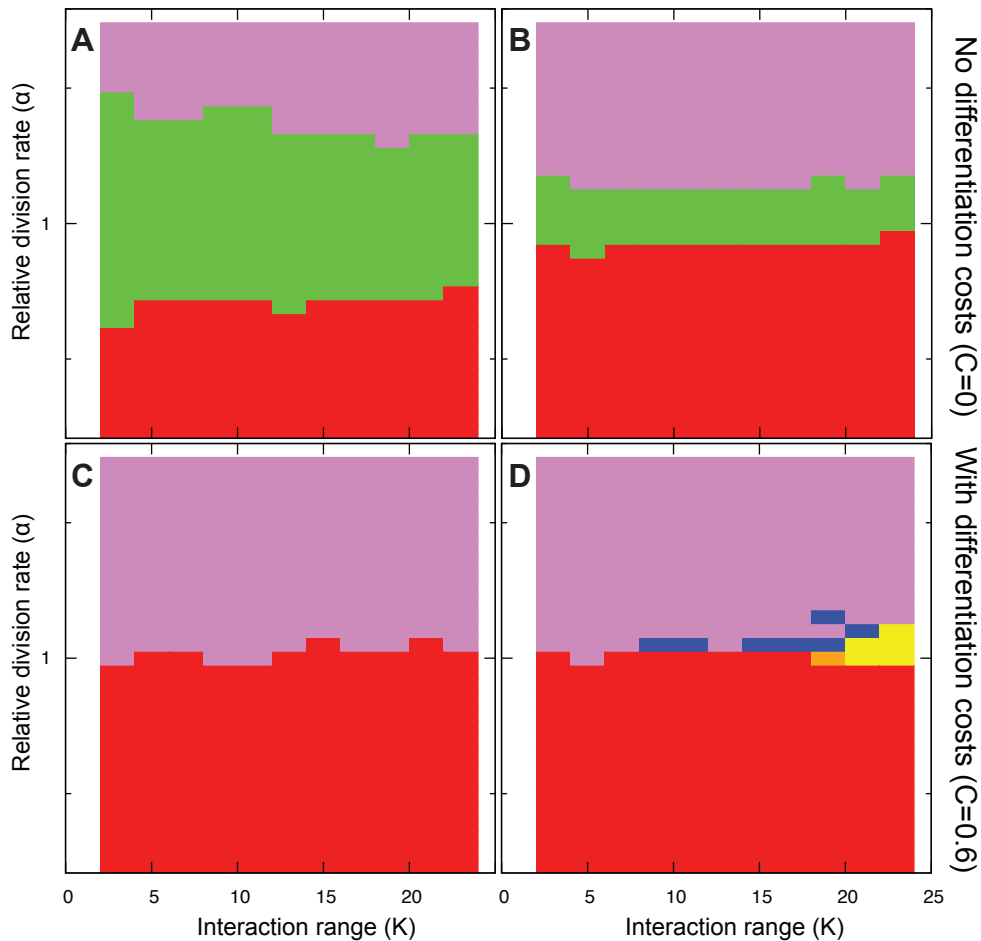

Supplement: Figure S4 — Most evolved developmental strategies in simulations where different cell types have symmetric fitnesses. Panels (a) and (c) show the results of the broken chain topology. Panels (b) and (d) show the results in the connected chain topology. The simulations were performed with varying cell interaction ranges and photosynthetic cell relative division rates , (a,b) with no differentiation costs () and (c,d) with differentiation costs (). Simulations were repeated 50 times for each parameter combination, with population sizes of 400. The color represents the most frequently evolved strategy coded according to Figure 4 in the main text. (PDF) [file pcbi.1002468.s004.pdf]

**A**

frequency

 $K=4$ 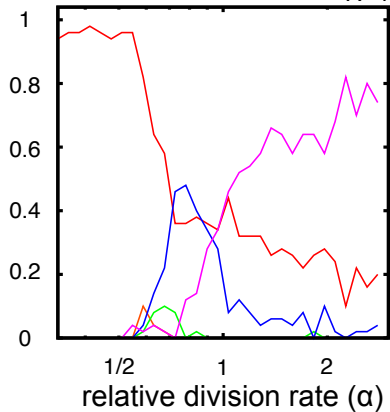**B** $K=12$ 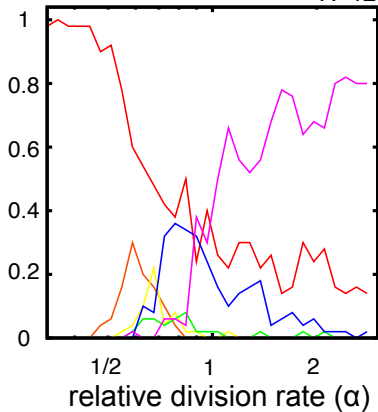**C** $K=24$ 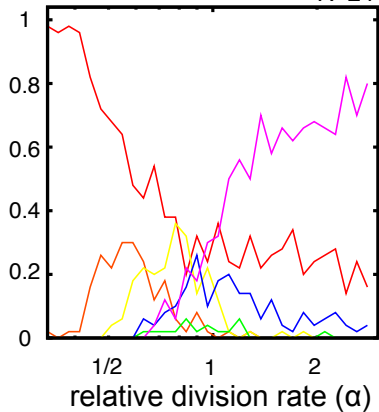

Supplement: Figure S5 — Model modification with a constant differentiation cost. Frequency of evolved developmental strategies using a constant differentiation cost in the connected chain topology. The plots show the frequency of evolution of each strategy with varying relative division rates (30 simulations per value). Each strategy is represented by a different colour according to the color key in Figure 2. The plots in the three different columns correspond to different interaction ranges (), as shown above each column. Simulations were performed with 200 cells over 5000 generations. (PDF) [file pcbi.1002468.s005.pdf]

**A**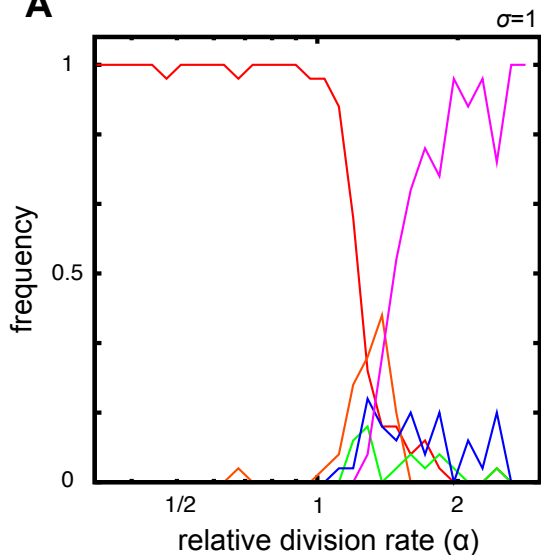**B**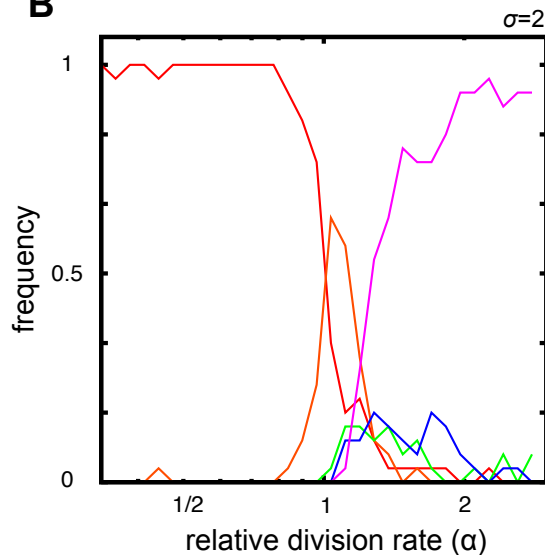**C**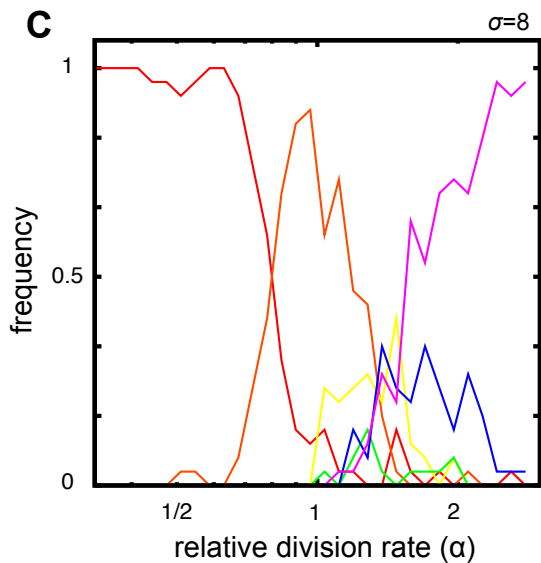**D**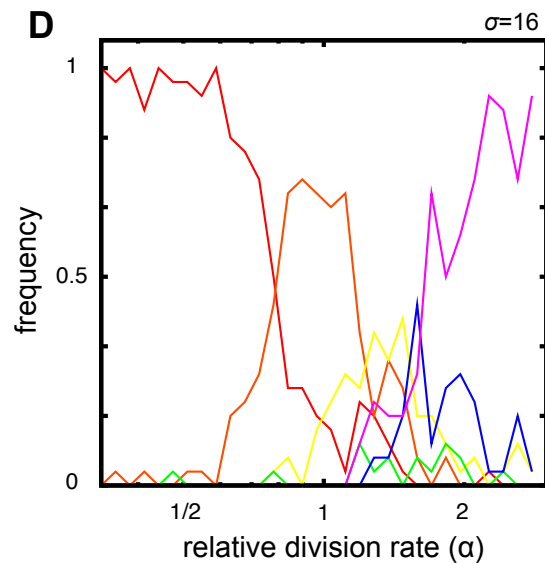

Supplement: Figure S6 — Model modification with a Gaussian function for interaction strength. Frequency of evolved developmental strategies using an interaction strength defined by a gaussian function with varying standard deviation in the connected chain topology. The plots show the frequency of evolution of each strategy with varying relative division rates (30 simulations per value). Each strategy is represented by a different color according to the color key in Figure 2. Simulations were performed with 200 cells over 5000 generations. (PDF) [file pcbi.1002468.s006.pdf]

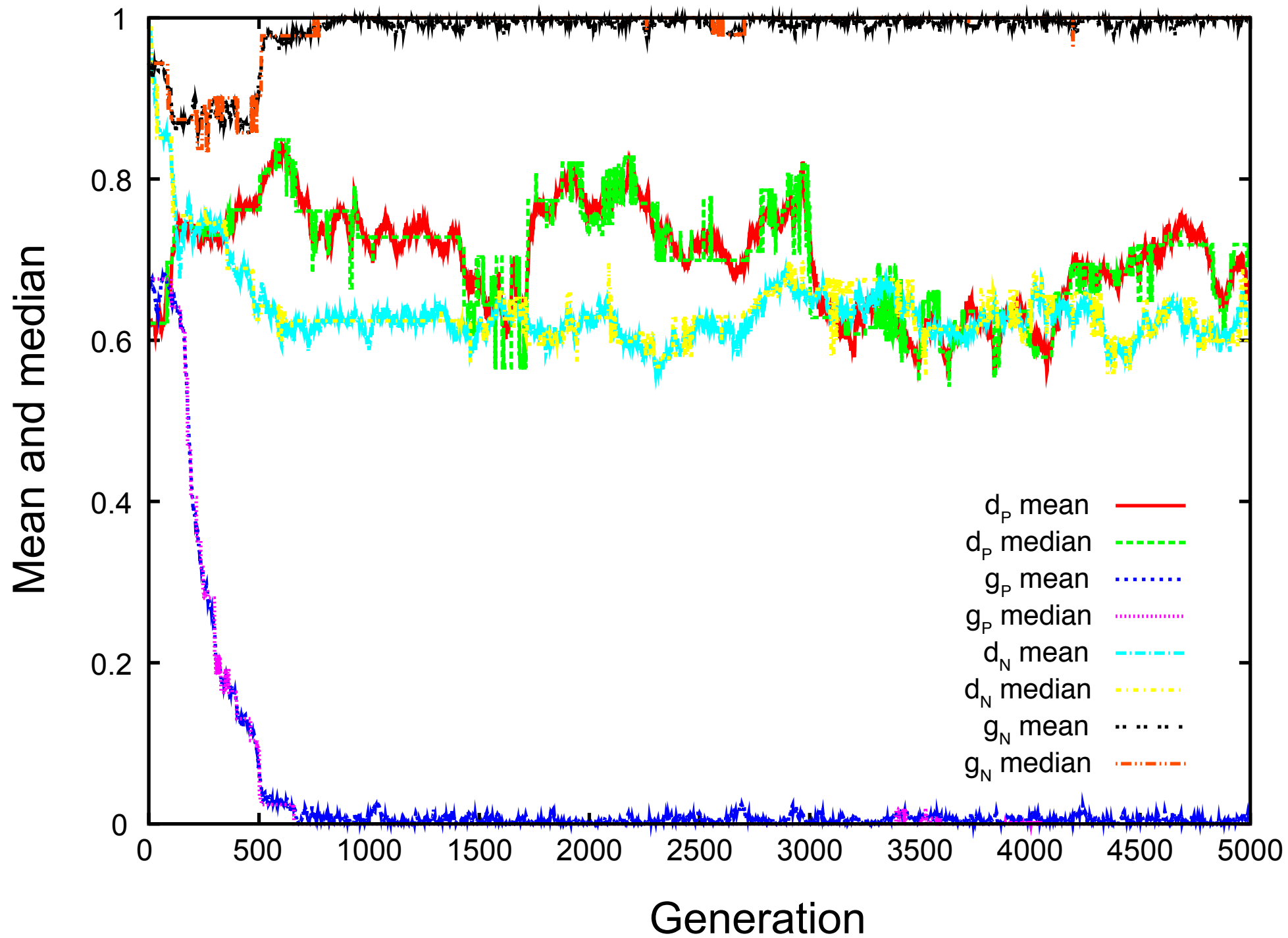

Supplement: Figure S7 — Comparison of mean and median of population trait values. Evolution of population trait means and medians (, , , ) of 200 cells over 5000 generations in the broken chain topology, with relative division rate and interaction range . (PDF) [file pcbi.1002468.s007.pdf]
