# Supplementary material for: Differences in Cell Division Rates Drive the Evolution of Terminal Differentiation in Microbes
Source: PLoS Comput Biol. 2012 Apr 12;8(4):e1002468. doi: 10.1371/journal.pcbi.1002468 (PMC3325182; doi:10.1371/journal.pcbi.1002468)
Supplement: Text S1 — Other model results, modifications and method details. (PDF) [file pcbi.1002468.s008.pdf]

Supplementary Information
Text S1

Differences in cell division rates drive the evolution of terminal differentiation in microbes

João F. Matias Rodrigues[*,a,b,c], Daniel J. Rankin[a,c], Valentina Rossetti[a], Andreas Wagner[a,c], and Homayoun C. Bagheri[a]

[a]Institute of Evolutionary Biology and Environmental Studies, University of Zurich, Zurich, Switzerland
[b]Institute of Molecular Life Sciences, University of Zurich, Zurich, Switzerland
[c]Swiss Institute of Bioinformatics, University of Zurich, Zurich, Switzerland

March 7, 2012

[*]To whom correspondence should be addressed. Email: joao.rodrigues@imls.uzh.ch, tel. +41 44 635 3148
[i]*Keywords: cyanobacteria, evolution of development, germline, soma, multicellularity, symbiosis, topology*
[ii]*Contribution type: article*

S1 Additional simulation results and methods

S1.1 Frequency of evolved developmental strategies for different filament topologies, interaction ranges, and differentiation costs

Figure S1 shows the plots of frequencies of the evolution of each developmental strategy when 50 stochastic simulations are carried out in the same conditions. Each plot shows how the frequencies change with varying relative division rates. The panels in row (A) in Figure S1 show the results in the case of the broken chain topology with no differentiation costs ($C = 0$). The panels in row (B) in Figure S1 show the broken chain topology with differentiation costs ($C = 0.2$). The panels in row (C) in Figure S1 show the case of the connected topology with differentiation costs ($C = 0.2$). Each of these four cases (Figure S1) were simulated with a varying cell interaction range ($K = 4, 12, 24$) which correspond to the plots on the left, middle, and right columns, respectively.

S1.2 Higher differentiation costs (C) and interaction ranges (K) favor symbiosis in the connected topology

In Figure S2 we examined the effect of increasing the differentiation costs ($C = 0.3$) in the connected topology. Here we compare these results to the case where the differentiation costs are lower ($C = 0.2$), as shown in Figure 4D in the main text. First, we observe that higher differentiation costs reduce the range of conditions un-

der which terminal differentiation without somatic division (violet and red) evolves. Instead, the symbiotic strategy (yellow), and terminal differentiation with somatic division, where the heterocyst is the germline (orange) evolve under a broader range of conditions. As a result, the dependency on interaction range becomes stronger, where increasing interaction ranges lead to a higher probability that one of these strategies evolves.

In Figure S3 we show the evolved developmental strategies at longer interaction ranges ($K = 24$ to $K = 40$) in the connected topology, in the cases of no differentiation costs ($C = 0$) and modest differentiation costs ($C = 0.2$). Longer interaction ranges in the case of no differentiation costs (Figure S3A) do not change the results seen at shorter interaction ranges (Figure 4B). In the case with differentiation costs, longer interaction ranges (Figure S3B) increase slightly the range of relative division rates under which symbiosis (yellow) and terminal differentiation with somatic division and a heterocyst germline (orange) occur when compared to shorter interaction ranges (Figure 4D).

S1.3 Qualitatively similar results are found in the symmetric model

In the model presented, the fitness of a photosynthetic cell and a nitrogen fixing cell is described by equations (5) and (9) in the main text. These equations describe a model that assumes that a nitrogen fixing cell can only perform its function when it is supplied with carbohydrates. One can also consider a different case where the ability to fix nitrogen is independent of the supply of carbohydrates. The latter

assumption leads to symmetric fitness functions for the photosynthetic and nitrogen fixing cells where:

$$f_{P_i} = \alpha min(g_{P_i}, R_{N_i}) + f_{base} \tag{S1}$$

$$f_{N_j} = min(g_{N_j}, R_{C_j}) + f_{base}. \tag{S2}$$

Because the photosynthetic cells are no longer the only source of energy in the system, both cells become equal partners needing the products from each other. In Figure S4 we show the developmental strategies that evolve in this symmetric model, in comparable conditions to the asymmetric model (Figure 4 in the main text). The results are qualitatively similar. However, in this case the evolved strategies symmetric around the relative cell division rate (α). In addition, we find that the symbiotic strategy is found to be restricted to a much narrower set of conditions characterized by high differentiation costs ($C = 0.6$).

S1.4 Bootstrap method to estimate confidence intervals in the frequency of evolving developmental strategies

To estimate the confidence intervals of the frequency of evolution of developmental strategies in sets of 50 simulations we used the bootstrap method. Briefly, the bootstrap method makes it possible to estimate the confidence intervals with fewer simulations through resampling. The confidence intervals shown in Figure 4 were obtained by generating 100 sets of 50 simulations drawing randomly from a pool of 500 previously simulated results.

S1.5 Gaussian interaction strength

We have simulated the interaction strength using a gaussian function $G_{\lambda,\sigma}(x)$ where λ represents the expected valued of the function, σ the standard deviation and x the distance between the interacting cells. In our simulations we used $\lambda = 0$ for a centered gaussian interaction. To implement the variation in the interaction strength with distance we first generalized the equations for R_{C_j} and R_{N_i} presented in the manuscript such that arbitrary strength coefficients s_{ij} define the interaction strength between cell i and j. The following set of equations,

$$R_{C_j} = \sum_{i}^{k_{P_j}} \frac{(1 - g_{P_i}) s_{ji} L}{\sum_l^{k_{N_i}} s_{il}}, \tag{S3}$$

$$R_{N_i} = \sum_{j}^{k_{N_i}} \frac{(1 - g_{N_j}) R_{C_j} s_{ij} V}{\sum_l^{k_{P_j}} s_{jl}}, \tag{S4}$$

will give the equations (9) and (10) presented in the manuscript for R_{C_j} and R_{N_i} when $s_{ij} = 1$ for all interacting photosynthetic cells giving carbohydrates to nitrogen fixing cells k_{P_j}, or nitrogen fixing cells giving fixed nitrogen to photosynthetic cells k_{N_i}.

If we replace the interaction strength coefficients s_{ij} with the gaussian function $G_\sigma(x_{ij})$ where x_{ij} represents the distance in neighborhood position from cell i to j, this will yield the formulas for R_{C_j} and R_{N_i} in the case of a gaussian interaction

strength between cells. Thus we obtain

$$R_{C_j} = \sum_{i}^{k_{P_j}} \frac{(1 - g_{P_i}) G_\sigma(x_{ji}) L}{\sum_l^{k_{N_i}} G_\sigma(x_{il})}, \tag{S5}$$

$$R_{N_i} = \sum_{j}^{k_{N_i}} \frac{(1 - g_{N_j}) R_{C_j} G_\sigma(x_{ij}) V}{\sum_l^{k_{P_j}} G_\sigma(x_{jl})}. \tag{S6}$$

The frequency of evolved developmental strategies is shown in Figure S6 in the case of the connected topology with differentiation cost $C = 0.2$. Due to computational constraints the implemented simulations only sum over the interaction strengths for the first 32 neighbors. This should not affect the results presented as it is well above the range of interaction range considered in the gaussian function $\sigma = 1, 2, 8, 16$ and therefore the coefficients further away should be negligible.
